# Supplementary material for: Exosome complex orchestrates developmental signaling to balance proliferation and differentiation during erythropoiesis
Source: eLife. 2016 Aug 20;5:e17877. doi: 10.7554/eLife.17877 (PMC5040589; doi:10.7554/eLife.17877)
Supplement: Supplementary file 1. — DOI: http://dx.doi.org/10.7554/eLife.17877.021 [file elife-17877-supp1.docx]

Supplementary File 1: Primers used for analysis of mRNA expression

| Locus | Forward | Reverse |
| --- | --- | --- |
| *Exosc8* | GGCTCTGCTCTCGTGAAGCT | TGGTGGTGCTGCAAATTCTG |
| *Exosc9* | CCCATTTGTGTCAGTTTTGCT | GTTCTTCACGTTCATTGGGGT |
| *Exosc3* | GCGTCTTTGTCTTACCTGGC | GACCACACACTGGCCATAGAT |
| *Kit* | AGCAATGGCCTCACGAGTTCTA | CCAGGAAAAGTTTGGCAGGAT |
| *Cdkn1b* | TTCGACGCCAGACGTAAACA | TCAGTGCTTATACAGGATGTCCA |
| *Ddit3* | AGCGACAGAGCCAGAATAACA | GCTTTCAGGTGTGGTGGTGTA |
| *Gadd45a* | AGACCGAAAGGATGGACACG | GGGTCTACGTTGAGCAGCTT |
| *Gas2l1* | GCCCAATGACCTTCGAAACC | ATCATGGGAAACTGGTCGGG |
| *Trp53Inp1* | AGTGAGGCGAGTTGTGGAAA | TGCCACACAGCAGTGAATGT |
| *Gata2* | GGCTCTACCACAAGATGAATGGA | AGGTGGTGGTTGTCGTCTGAC |
| *Vim* | CCAGAGAGAGGAAGCCGAAA | CGTGCCAGAGAAGCATTGTC |
| *Hdc* | ACCTCCGACATGCCAACTCT | CCGAATCACAAACCACAGCTT |
| *Samd14* | TGTAGGGCGTGTTCAGAGATAGAG | TGACCAGCACCCAAGAAGCT |
