# Supplementary material for: Exosome complex orchestrates developmental signaling to balance proliferation and differentiation during erythropoiesis
Source: eLife. 2016 Aug 20;5:e17877. doi: 10.7554/eLife.17877 (PMC5040589; doi:10.7554/eLife.17877)
Supplement: Supplementary file 2. — DOI: http://dx.doi.org/10.7554/eLife.17877.022 [file elife-17877-supp2.docx]

Supplementary File 2: Primers used for ChIP analysis

| Locus | Location relative to promoter (kb) | Forward | Reverse |
| --- | --- | --- | --- |
| *Krt5* | -0.025 | TGCCCAACCCACTTCTAAGG | AGGTCAGCGGCGTTCAAC |
| *MyoD* | -1.1 | CCAGATCTCAGTGCTGCAGG | CCGCTTGCATAGCATAACCAG |
|  | -0.07 | GGGTAGAGGACAGCCGGTGT | GTACAATGACAAAGGTTCTGTGGGT |
| *Slc4a1* | -0.2 | GGGTGCCCCATAAACAGAGTT | TGAAGGGCCCAAAGACCTTAG |
| *Alas2* | -0.07 | TACCCCAGAGGGAGTAGAGGAAG | AGGTATCTCAGGCCTCTGCTAAAG |
| *RpII215* | -0.01 | GCGAATCTATAAAGGGCGTCACT | TCGGCGCTTCTGAGGAGA |
| *Hbb-b1* | -0.07 | CAGGGAGAAATATGCTTGTCATCA | GTGAGCAGATTGGCCCTTACC |
| *Kit* | -114 | GCACACAGGACCTGACTCCA | GTTCTGAGATGCGGTTGCTG |
|  | -0.17 | ACTGAAGGACCACCGATGGA | TGCCCTCTAAGACCAGGAGC |
|  | +0.2 | GATCTGCTCTGCGTCCTGTT | ATGGGAAAAGCCAACAGCTA |
|  | +5 | GGCTGGAAACCACTGCCTTA | AGCCTTGCCTGTGCTTAAAGC |
|  | +35 | TCCCACTGTGAAGGAAGGAC | GCACTCACCTGAGGGTTCAT |
|  | +57 | TTGGGCATATCACTGCGTTA | GCACCTCCTTGCCTGTTAAG |
|  | +58 | GGAGGAGTTAGGGAATATGTCGATAG | GCAGTTCTCCAGGTTGAGTCAGA |
|  | +75 | CCTCAGCCCATTTGAACAGT | AGCCAGCAATACCCTTCCTT |
|  | +80.5 | TCCATGTCAGAGTGGCTTTG | ACAACTCACCCACACGCATA |
|  | +80.8 | CTCACATAGCAGGGAGCACA | ATGCAGGGAAACTGGAGAGA |
